# Effect of nanoclay orientation on oxygen barrier properties of LbL nanocomposite coated films†

**DOI:** 10.1039/c8ra09522a

**Published:** 2019-01-11

**Authors:** Fatma Ben Dhieb, Ebrahim Jalali Dil, Seyyed H. Tabatabaei, Frej Mighri, Abdellah Ajji

**Affiliations:** 3SPack NSERC-Industry Chair, CREPEC, Chemical Engineering Department, Polytechnique Montreal C.P. 6079, Succ. Centre Ville Montreal QC Canada H3C 3A7 abdellah.ajji@polymtl.ca; ProAmpac Terrebonne QC Canada J6Y 1V2; CREPEC, Chemical Engineering Department, Laval University Quebec QC Canada

## Abstract

Layer by layer (LbL) film deposition is an efficient technique used to produce thin coatings with high gas barrier properties. In this study, multilayer composite coatings with hydrogen bonding inter-layer interactions were deposited by LbL on a PET substrate, with an alternate deposition of a nanoclay layer and different intercalating polymers layers, namely chitosan (CS), polyethylene oxide (PEO), polyvinylpyrrolidone (PVP) and polyvinyl alcohol (PVA). The investigated coatings had two different structures, quadlayers and bilayers which are different in the number of layers in the repetitive unit (four and two respectively). The alignment of nanoclay platelets and the extent of their intercalation were studied using Fourier transform infrared (FTIR) spectroscopy and X-ray diffraction (XRD). The results showed that the dispersion level and the orientation of nanoclay particles depend considerably on the molecular structure of intercalating polymers and their interactions with nanoclay. An oxygen permeability model, specific to high filler loading composites, was then developed by considering only the aspect ratio and the volume fraction of the nanoparticles.

## Abstract 2

Quantify clay orientation in a layer by layer deposited films. Demonstrate the importance of interfacial region in a high clay loading nanocomposite film, in decreasing permeability.

## Graphical abstract

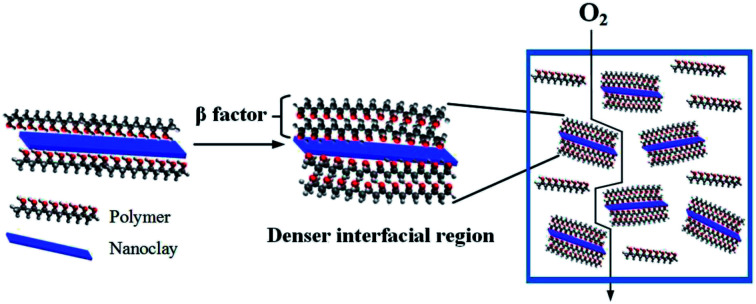

## 1 Introduction

Given their light weight, high chemical resistance and low interaction with food, polymers have been considered good candidates for packaging and their wide variety have allowed for a large spectrum of properties. Nevertheless, the constant demand for better oxygen barrier packaging motivated research toward improving the barrier properties of polymers. This depends mainly on the diffusivity of gas molecules and their solubility in the polymer.^1^

Incorporation of additives such as nanoclay is among the investigated alternatives to improve the properties such as the oxygen barrier.^2–4^ The impermeable structure of clay coupled with a large aspect ratio, increase tortuosity along the path of a gas molecule through the polymer, which consequently reduces gas permeability. The relative permeability is inversely proportional to tortuosity, *τ*.^5^1
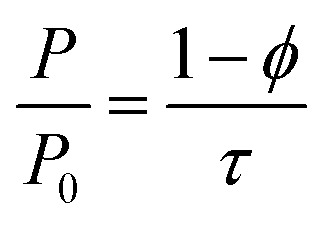
where, *P*_0_ is the permeability of the non-filled polymer and *ϕ* is the volume fraction of the filler. Tortuosity can vary considerably with the aspect ratio of nanoclay, its volume fraction, intercalation and orientation. This latter trait has been extensively investigated for melt processed films.^6–8^ In addition to the direct effect of nanoclay on tortuosity, it can also indirectly affect the tortuosity by altering the crystallinity and orientation of polymer crystallites.^9^ For instance, a preferred orientation of nanoclay and crystallites along the machine direction have been previously reported in the literature and is attributed to the shear stress in the die and the large elongational stress at the die exit.^10,11^ Therefore, it can be seen that, besides dispersion, controlling nanoclay orientation is critical to the final nanocomposites properties.^12^

Recently layer by layer (LbL) coating has emerged as a new method to produce much thinner nanocomposite coatings with high oxygen barrier properties,^13,14^ since the lack of clay exfoliation and its aggregation, hinder the use of clay with conventional processes, *e.g.* extrusion.^15^ High clay concentration can be achieved with this technique, enabling, thus, a good oxygen barrier.^16–22^ Many types of clay are used for coating (hectorite, synthetic mica, vermiculite (VMT), montmorillonite (MMT)…) as they improve gas barrier and mechanical properties. MMT, for instance was extensively studied for LbL coatings. With its charged oxygens and hydroxyl groups on the surface it can establish hydrogen bonds and electrostatic interactions with polymers. Among different aspects of LbL nanocomposites, the effects of nanoclay dispersion and *d*-spacing on oxygen barrier properties, mechanical properties and transparency are the most commonly studied aspects in the literature. LbL is a simple technique relying on interaction between layers. This could be based on electrostatic interactions between charged polymers, hydrogen bonding, van der Waals forces or hydrophobic interactions.^23^ Among them, the most studied ones are electrostatic^18^ and hydrogen bonding^24,25^ interactions. Properties of the LbL assembled films depends considerably on the type of these interactions. For electrostatic interactions based coatings, polymers such as poly(acrylic acid), PAA, polyethyleneimine, PEI,^26,27^ polyvinylamine, PVAm,^18^ polyacrylamide, PAM^22^ have been used and resulted in very dense films. Hydrogen bonding interactions, on the other hand, allow a more flexible structure that can withstand mechanical strains while maintaining their oxygen barrier.^28^ Chitosan (CS), polyethylene oxide (PEO), polyvinylpyrrolidone, PVP, and polyvinyl alcohol (PVA) are among the most common polymers used for hydrogen bonding based coatings which will be used in this study.

Chitosan is from a renewable source, recognized as having good barrier properties and forms film easily (from solutions). PVA has a considerable density of functional groups and establishes strong hydrogen bonds. Contrary to PVA, PVP has a rigid structure allowing a more linear growth of LbL film and, similar to chitosan, it can readily form films. PEO has a linear, non-branched structure with different functional group than PVA and PVP. These differences allow studying the effect of molecular structures on the properties of the film samples. The operating parameters for the LbL technique have been extensively studied to determine their effect on the resulting film properties. For instance, the pH,^19^ the deposition time^17^ and the application of different procedures such as removing the drying step^29^ were key factors in improving the film properties by tuning the density and the behavior of the resulting layers.

To characterize these properties, many methods have been adopted, particularly thermal analysis to determine the thermal transition temperature,^25,30^ ellipsometry to measure the layers thickness,^16,21,31,32^ quartz crystal microbalance, QCM-d, to track the mass change with each layer^33,34^ and gas transmission measurement to evaluate the film permeability.^35^

As LbL technique allows achieving good dispersion even at high nanoclay contents, nanoclay coatings produced by this technique show good oxygen barrier properties.^16–22^ It is known that the tortuosity of the path of the gas molecule is strongly related to nanoclay orientation in the deposited layers. However, despite the significant potential of LbL coatings, there is little information about the clay orientation.^15^

Since nanocomposites properties, such as oxygen permeability, are considerably dependent on tortuosity, the study of clay orientation and intercalation will be carried out for two types of assemblies, bilayer and quadlayer. For the selected coatings, orientation will be determined using FTIR measurements and by using two different quantification methods. As the used polymers (PVA, PEO, PVP and CS) have different potentials to establish hydrogen bonding interactions,^35–40^ the obtained results will allow to investigate the effect of different levels of interactions of polymers and nanoclay through the study of the properties of the coatings such as density and oxygen permeability as well as nanoclay dispersion. The experimental results will be then compared with permeability models and a modification of some of those models will be discussed.

## 2 Experimental

### 2.1. Materials

**Fig. 1:**
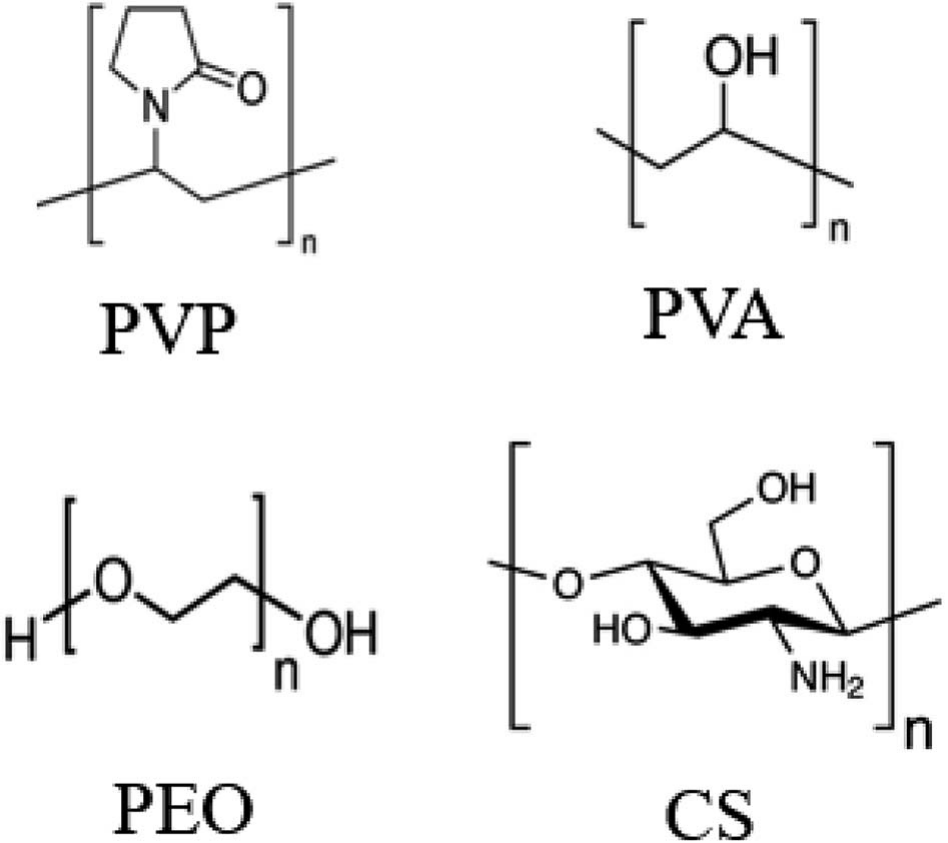
Molecular structures of PVP, PVA, PEO and CS.

Natural sodium montmorillonite (MMT) platelets (Cloisite NA^+^), nanoclay, with density of 2.86 g cm^−3^ were supplied by BYK (Gonzales, Texas, United States) and used as received. Chitosan (*M*_w_ = 150 000 g mol^−1^), PEO (*M*_w_ = 4 000 000 g mol^−1^), PVP (*M*_w_ = 360 000 g mol^−1^) and PVA (*M*_w_ = 140 000–186 000 g mol^−1^) (Fig. 1) were supplied by Sigma Aldrich (Saint Louis, Missouri, USA). Different substrates were used according to the characterization technique or the type of test conducted: silicon platelets were used for XRD and profilometry characterization and were purchased from EL-Cat Inc. (Ridgefield Park, New Jersey, United States). Polyethylene terephthalate (PET), 16 μm thickness, (supplied by ProAmpac (Terrebonne, QC, Canada)) was mainly used for permeability tests and AFM characterization and low density polyethylene (LDPE) (supplied also by ProAmpac) served as substrate only for FTIR analysis.

### 2.2. Preparation of thin coatings

All the solutions were prepared with deionized (DI) water and had the same concentration of 0.1% wt except for MMT (0.5% wt). PVA solution was heated at 80 °C for 2 hours and the pH of chitosan solution was adjusted to 6 by adding acetic acid and 1 molar sodium hydroxide (NaOH). In order to generate a primer layer, all cleaned substrates were initially dipped into a PEI solution (0.6% in DI water) for 20 min and then rinsed with DI water. The coating deposition of each layer consists of three steps, dipping in polymer or MMT solution, rinsing with DI water and drying. Fig. S1† shows schematics of the coating cycle for a quadlayer and bilayer assemblies. For the first deposited bilayer or quadlayer, dipping was done for 5 min and then the sample was rinsed for 1 min. For the following layers, dipping and rinsing times were reduced to 1 min and 30 s, respectively.

### 2.3. Characterization of coatings

#### 2.3.1 Fourier transform infrared spectroscopy (FT**I**R)

In order to investigate orientation of the nanoclay platelets in the thin coatings, infrared analysis was elaborated using a Spectrum 65 FTIR spectrometer from PerkinElmer (Waltham, MA) with a resolution of 4 cm^−1^ and a 32 scans accumulation within wavenumber range of 900 to 1200 cm^−1^. All experiments were performed using a Spectra-Tech zinc selenide wire grid polarizer from Thermo Electron Corp. Three types of spectra were recorded with the polarizer; in the vertical machine direction, *S*_M_, in the horizontal transverse direction, *S*_T_ and in the horizontal direction with a tilted film at an angle *φ* with respect to the machine direction (MD), *S*_NT_. In order to avoid peaks saturation and overlapping, LDPE was used as substrate. Montmorillonite has four Si–O stretching bands around 1080, 1025, 1048,1120 cm^−1^.^7^ The peaks at 1025, 1048 and 1120 cm^−1^ are associated with oxygen at the surface of the clay platelets (basal oxygen) and the peak at 1080 cm^−1^ corresponds to apical oxygen. This latter peak is the oxygen at the internal edge of the tetrahedral sheet, linked to Aluminum and having its Si–O bond perpendicular to platelet plane.^41^ The spectrum in the normal direction, *S*_N_, can be calculated with the following equation.^42^2
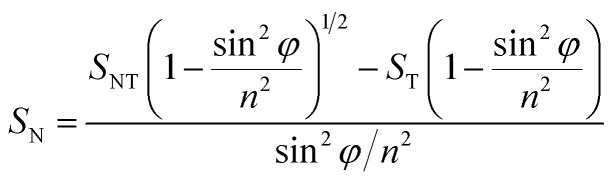


Considering *φ* equal to 45° and *n*, the refractive index of Montmorillonite equal to 1.503,^43^ the equation can then be simplified to:

3 *S*_N_ = 3.968*S*_NT_ − 3.5*S*_T_

The structurally independent spectrum *S*_0_, represent the arithmetic average of the three spectra, *S*_M_, *S*_N_ and *S*_T_.

Since orientation of clay platelets can be characterized by the orientation of their plane normal, these can be calculated with the following Herman's orientation function:^7^4
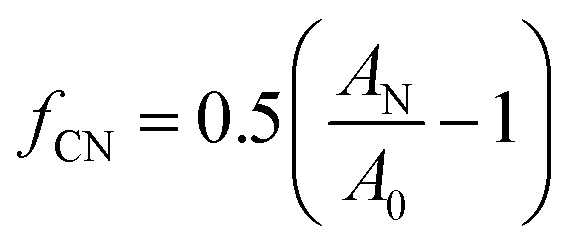
where, *A*_N_ and *A*_0_ are the band intensities in the *S*_N_ and *S*_0_ spectra corresponding to the peaks whose vibrational transition moment lies along the *c*-axis, normal to the platelets plane. The ratio 
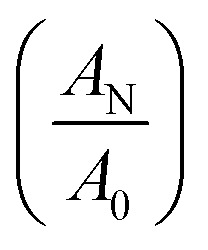
 is referred as dichroic ratio, *D*.

#### 2.3.2 X-ray diffraction spectroscopy

A Philips X'pert apparatus was used to carry out wide angle X-ray diffraction (WAXD) spectroscopy. Measurements were done using a copper CuKα radiation source (*λ* = 1.54056 Å). Coatings deposited on silicon wafers were scanned from 2 to 10 degrees at a rate of 0.02° s^−1^. The MMT interlayer spacing (*d*_001_) was determined using the Bragg's law and the diffraction angle at the maximum intensity peak in XRD patterns.

#### 2.3.3 Oxygen transmission rate

The permeability to oxygen was measured *via* a MOCON OXTRAN 2/21 (Minneapolis, USA) at 25 °C, at 0% relative humidity, and 1 atm pressure, in accordance with the ASTM D-3985-81.

#### 2.3.4 Morphology of coatings

Coatings deposited on silicon substrate were tested using a Dektak 3030 profilometer to determine their average thickness and calculate their roughness.

In order to prepare the surface for AFM analysis, PCL was first melted at 80 °C and then the film was embedded into the molten PCL. The sample was cooled to room temperature and crafted in a pyramid shape tip using a razor blade. The sample was then microtomed using a cryo-microtome (Leica-Jung RM 2065) operated at −170 °C. The morphology of the cross-section of coated layers was then examined using an Atomic Force Microscopy (AFM) machine (Nanoscope V Dimension Icon/Fastscan AFM, Bruker, USA) operated in tapping mode in air. All AFM images were acquired using Intermittent Peak Force tapping™ using 125 μm TESPA-V2 Air probes with tip radius of 8 nm. Due to the difference in the modulus of the materials in the molded sample, tapping phase mode was used in the analysis of the nanostructure of the coated layer.

The thickness of the coated layer and the size of nanostructures in the layer were determined using the free ImageJ software. The average values are reported as XX ± YY where XX is the average value and YY shows the standard deviation.

## 3 Results and discussion

### 3.1. Properties of clay

#### 3.1.1 Orientation

The fitting method was used to determine the orientation of the clay platelets. The dichroic ratio (*D*), was calculated with the area of the peaks after deconvolution, as illustrated in Fig. S2† for a PVA quadlayer. The dichroic ratio was based on the two peaks area of the apical oxygen at the wavenumber of 1080 cm^−1^ and was equal to the ratio of their sum in the *S*_N_ spectrum on their sum in the *S*_0_ spectrum. This value is proportional to the orientation of clay perpendicularly to the normal vector of the coating. The chosen function to fit the peaks was PearsonVII, as it confers an intermediate shape between Lorentzian and Gaussian, adjustable with a shape factor parameter. Two of the four orientation peaks corresponding to Si–O stretching were split in two peaks, according to Cole *et al.*^6^ The MD and transverse direction (TD) spectra were perfectly superposed because of a similar orientation in both directions.

To corroborate these results, a spectral subtraction method, Fig. S3,† was used. The dichroic ratio corresponds to the subtraction factor necessary to eliminate the apical oxygen peak (1080 cm^−1^).^7^

**Fig. 2:**
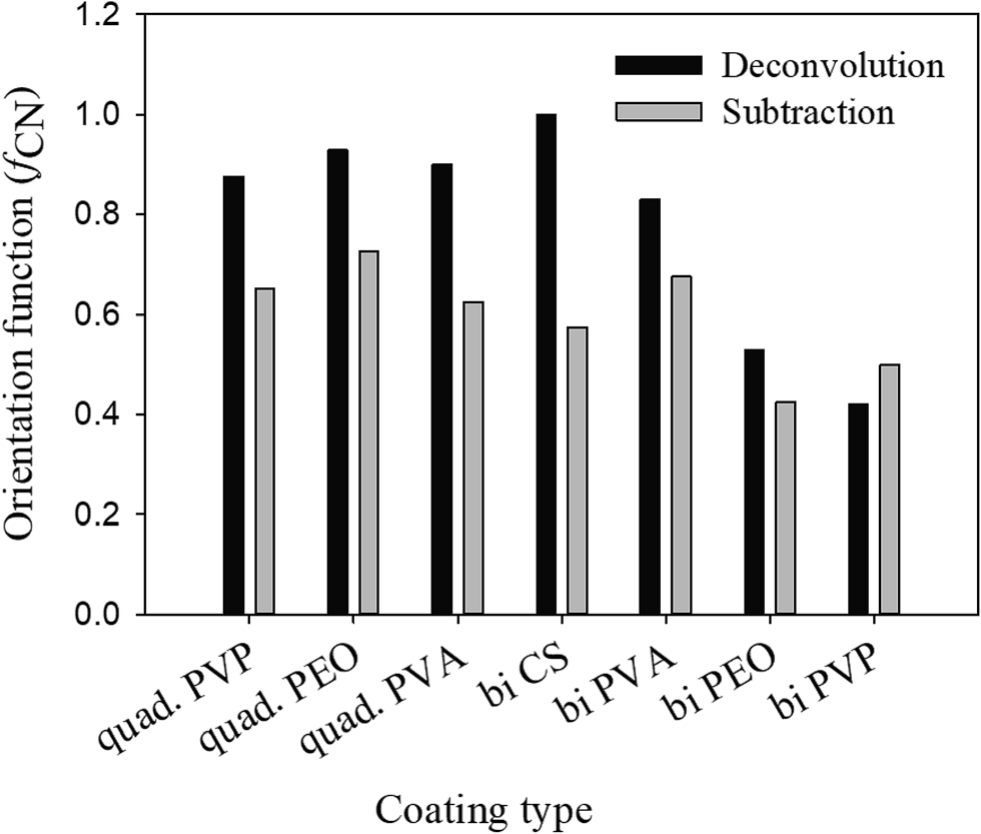
Herman orientation function, *f*_CN_, determined by subtraction and deconvolution.

The obtained values of orientation function with both methods are not the same for most of the assemblies, as shown in Fig. 2, probably due to uncertainties in the deconvolution procedure, but are sufficiently close to ascertain the range of the results obtained.

**Fig. 3:**
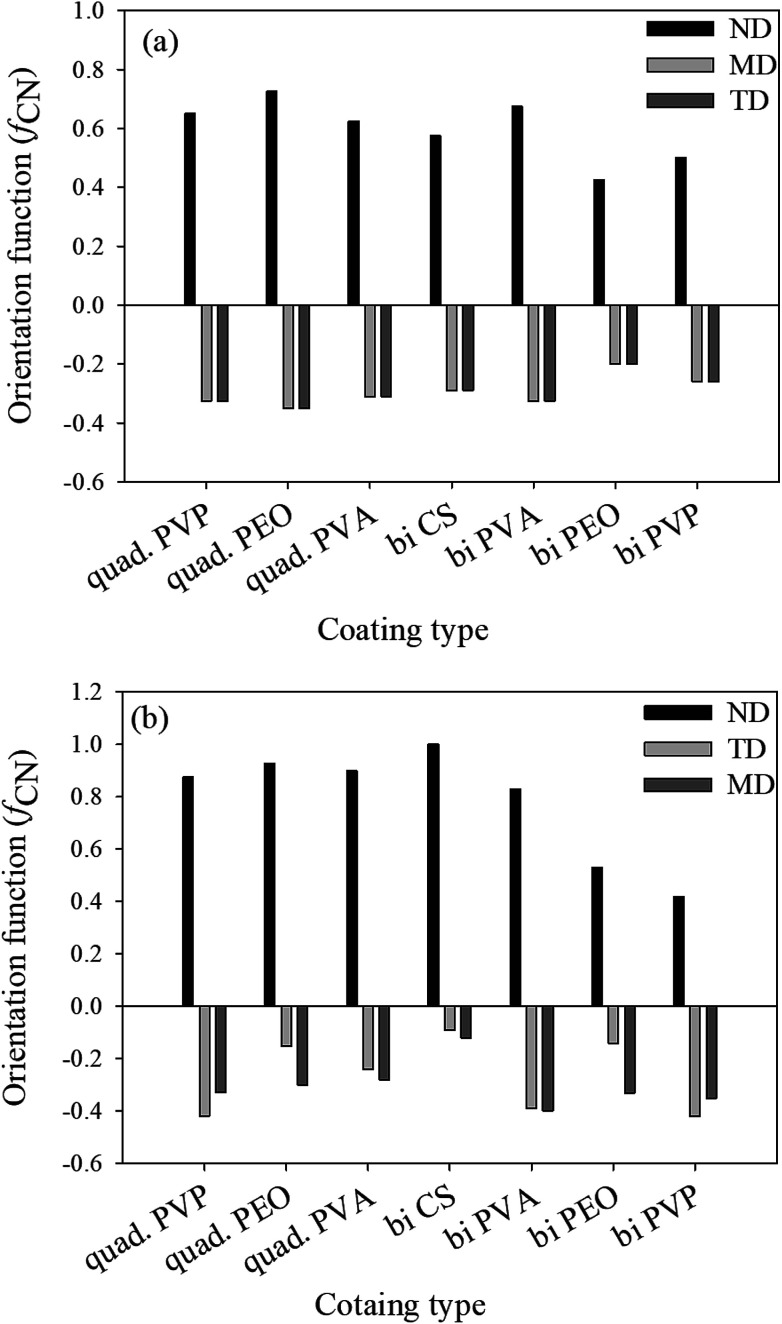
Herman orientation functions of the studied assemblies determined by subtraction (a) and deconvolution (b).

It's known that the sum of orientation functions in different directions should be zero.^7^ In the case of the deconvolution method, this sum is considerably greater than zero due to the effect of different parameters such as shape factor, peak width and center upon the final results. On the other hand, the obtained results from the subtraction method show a sum close or equal to zero for all the assemblies (Fig. 3). Based on the aforementioned discussion, the subtraction method will be used in the following discussions for the rest of the paper.

It is well known that shear forces during melt processing of nanocomposites tend to orient clay platelets in the machine direction.^7^. This, however, is not the case for the coatings deposited with the LbL method. For this technique, clay platelets interact freely in solution with the polymers deposited on the substrate. Bearing in mind that MMT platelets establish hydrogen bonding as well as the electrostatic interactions, due to its hydroxyl groups and oxygen, the main difference in clay orientation for the studied assemblies would be at the level of the established hydrogen bonds, in their strength and density. When compared with the orientation function for the different polymers (PVP, PEO and PVA) and the two types of assemblies, bilayer and quadlayer, a similar trend is observed for each polymer (Fig. 3) with an improvement of orientation for the quadlayer. For the bilayers assemblies, Hermans orientation function has a higher value in the case of the PVA bilayers followed by CS, PVP and PEO bilayers. This can be interpreted by a high affinity of PVA and MMT resulting in a denser structure constraining thus MMT to a certain orientation. Contrary to PEO, CS and PVP have cyclic groups in their structure, stiff enough to avoid entanglement and deposit in an orderly manner, hence constraining MMT to an ordered deposition as well. This CS structure explains the improvement of orientation for the quadlayers structure compare to bilayers. As CS layers alternate the PVA and MMT layers in the PVA quadlayers, the high interaction of PVA with MMT is disrupted causing a slight decrease in orientation.

#### 3.1.2 Nanoclay dispersion

**Fig. 4:**
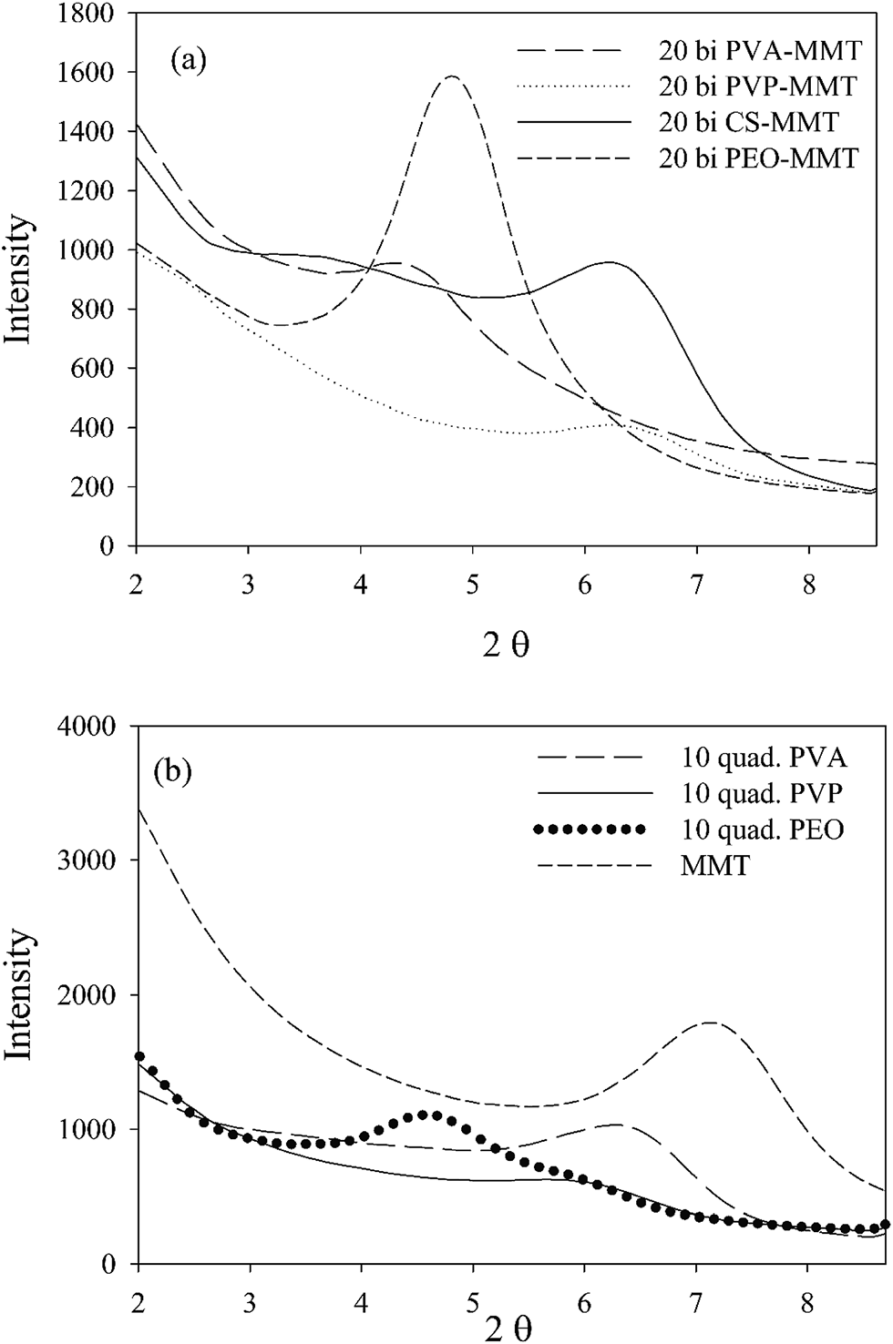
XRD patterns of the studied assemblies, (a) bilayers and (b) quadlayers.

Unlike orientation, nanoclay dispersion is extensively studied in nanocomposites, due to its significant role on the dispersion state of nanoparticles. Interlayer spacing of nanoclay has been studied using WAXD spectroscopy of nanocomposites and the neat nanoparticles. As it can be seen in Fig. 4, the obtained results indicate that MMT interlayer spacing (1.23 nm) increased in all studied assemblies, however, the intercalation level of nanoclay tactoids depends considerably on the polymer type and assembly.

The affinity between polymers and nanoclay is better understood with nanoclay intercalation in the bilayers.

As discussed, PVP, PEO and PVA established hydrogen bonds with MMT implying that their intercalation in the clay interlayer spacing depends on the extent of hydrogen bonding. Chitosan and PVP bilayers have the lowest intercalation, meaning a more stacked clay platelets than PEO and PVA bilayers. This weak intercalation may be explained by the presence of cyclic groups in both polymers which imparts rigidity to the polymer chain. The better intercalation in the PVA bilayers compare with the PEO is due to the higher reactivity of its functional groups.

Considering that the rigidity of chitosan hinders its diffusion,^4^ one can infer that nanoclay intercalation, for a quadlayer assembly, depends mainly on the diffusion of polymers through the chitosan layers. As MMT is trapped between chitosan layers, nanoclay intercalation should not be much different for the three quadlayers. However, it can be seen that the level of nanoclay intercalation depends on the polymer type in the three quadlayers assemblies. This supports previous studies suggesting that LbL assemblies have interpenetrated structures.^44–47^

PEO and PVP quadlayers have almost the same intercalation of the bilayers with a slight increase, whereas PVA quadlayers have a decrease in the interlayer spacing compare to the bilayers due to the interference of CS in the interaction with MMT (Table 1).

**Table 1:** Physical properties and crystallography of the studied assemblies

Properties		Multilayers						
		PEO	PVA	PVP	CS	PVA	PVP	PEO
		6 quadlayers			12 bilayers			
Orientation function (*f*_CN_)		0.725	0.625	0.65	0.575	0.675	0.5	0.425
Thickness (μm)		0.266	0.236	0.309	0.33	0.37	0.5	0.48
Crystallo.	2 theta	4.63	6.46	5.97	6.47	4.44	6.43	4.83
	*d*-spacing (Å)	19	13.67	14.78	13.64	19.89	13.73	18.26

The main difference between quadlayer and bilayer structures is the interaction between the polymer layers. In a bilayer, the polymer interacts mainly with clay whereas in a quadlayer, the interaction between adjacent polymers is the predominant one. A study of the morphology of the assemblies could shed more light on those interactions.

#### 3.1.3 Coating morphology

The thickness of the assemblies was measured by profilometry, Fig. S4.† For the three quadlayers, the increase in the thickness is linear related to the number of layers. However, the slope of the increase in thickness depends on the type of polymer. PVA resulted in a thinner coating, most probably due to a higher density of established hydrogen bonding with CS and MMT, while PVP led to thicker coatings which, could be attributed to a considerable fraction of free volume in the coating.

Table 1 summarises some physical properties and crystallography of the studied assemblies measured by FTIR, profilometry and XRD. For the same number of layers, the bilayers are thicker which is probably due to the higher nanoclay content of these structures. Indeed, with lower amount of nanoclay, the quadlayers have more interaction and inter-diffusion between the polymer layers, resulting in a thinner structure compare to bilayer assemblies.

**Fig. 5:**
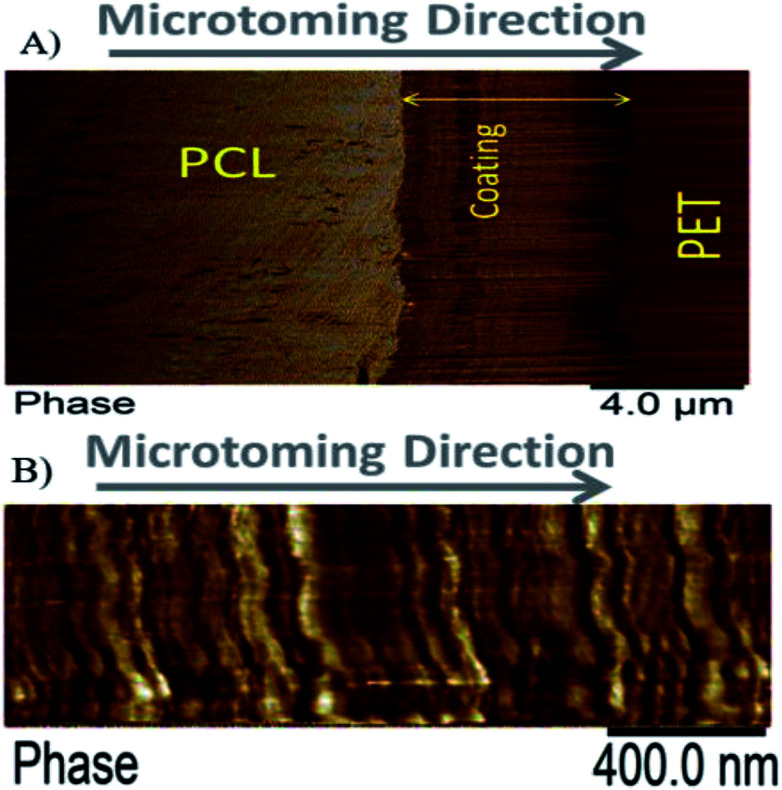
Atomic Force Microscopy (AFM) images of a PVA quadlayer coating. The cross section scanned at low (A) and high (B) magnification illustrates the layered structure of the coating.

The nanostructure of the coating was examined using AFM imaging. Fig. 5A and B show the typical nanostructure of a PVA quadlayer coating.

The periodic multilayer structure is composed of two different materials shown as a darker and a brighter phase, Fig. 5. The darker phase in the AFM image indicates a longer contact time between the tip and the surface which could be due to the softness or higher level of interactions of the surface with the tip. As the AFM tip used in this study is made of silicon, a better interaction between MMT nanoparticles and the tip is expected.

**Fig. 6:**
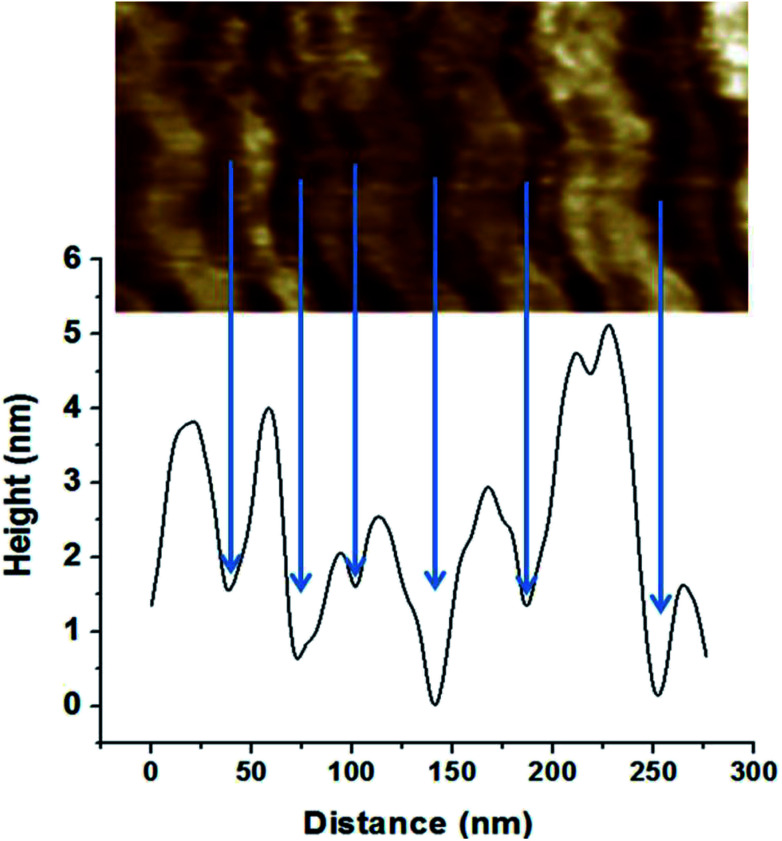
Height profile of the coating.

The nature of the darker phase can also be examined using the height profile over different regions of coated layer, Fig. 6. The results indicate that the darker phase has always a lower height compared with the other phase. Considering that the thermal expansion coefficient of silica based material is at least ten times lower than that of polymers such as PVA,^48,49^ both lower height and darker color leads to the conclusion that the darker phase should be the nanoclay layer.

Image analysis results indicate a layer thickness of 31 ± 11 nm for the darker phase and 52 ± 25 nm for the brighter phase. The greater standard deviation of the brighter phase indicates a broader distribution for the thickness of this layer. During coating preparation, a four-layered sequence was deposited, CS/PVA/CS/MMT but, only two layers can be observed with AFM. These results further corroborate our previous results indicating PVA diffusion through the chitosan layer. As chitosan and PVA are miscible^50^ and PVA layer is trapped between chitosan layers, only one phase represents all of the polymers' layers.

### 3.2. Permeability to oxygen

**Fig. 7:**
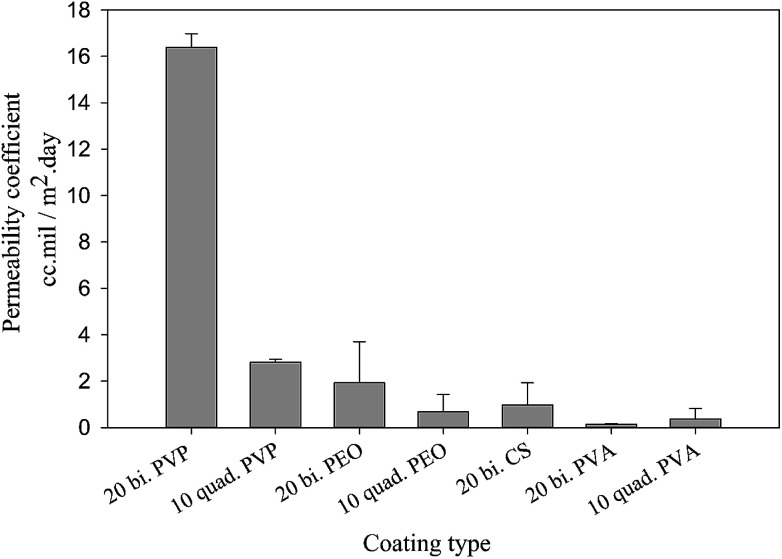
Oxygen permeability for bilayer and quadlayer assemblies.

The better understanding of nanoclay properties as well as polymer interactions in the prepared assemblies allow a much deeper interpretation of oxygen permeability results. As discussed previously, the main difference between bilayer and quadlayer assemblies is the polymers interaction in the quadlayers. The importance of this interaction is clearly perceived through the difference in permeability between the two types of assemblies (Fig. 7). Both types of coatings reduce considerably the neat PET permeability, 156 cm^3^ mil/m^2^ day.

In contrast with PVA, both PVP and PEO quadlayer assemblies have four times less permeability than bilayers; this result correlates well with those of Priolo and *et al.*^16^ and further supports the idea of a better oxygen barrier with higher spacing between clay layers. Considering that bilayer coatings are thicker than quadlayers, as shown in Table 1, the latter have clearly better intermolecular interaction due to the presence of CS layers that allow more hydrogen bonding in the films, resulting in a better oxygen barrier.

The PVA bilayer, however, has a better barrier than the quadlayer. PVA is known for its significant interaction with nanoclay, resulting in thin coatings with appreciable mechanical properties.^38,51^ The incorporation of CS can create more free volume in the coating since it has a rigid structure, which, even with good electrostatic interactions and hydrogen bonding, results in more free volume compare with the PVA-MMT bilayers. This would explain the lower intercalation and orientation in the case of PVA quadlayers compare to the bilayers.

**Fig. 8:**
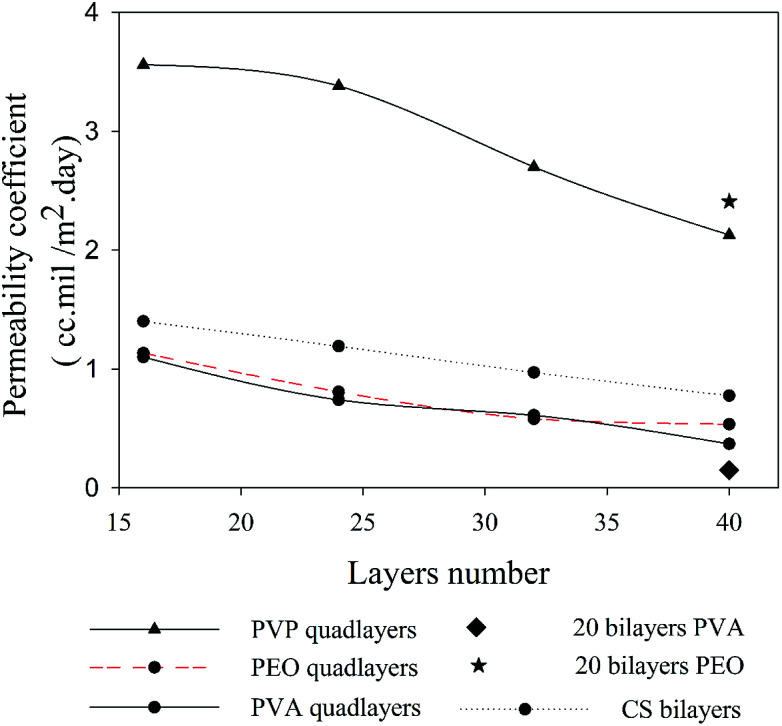
Oxygen permeability as a function of the number of deposited layers for four types of assemblies.

Despite the better intercalation with PVP compare to PVA for the quadlayers assemblies, the permeability to oxygen of the PVP quadlayers is much higher, Fig. 8. This implies that the intermolecular interaction has more impact on the oxygen barrier than clay intercalation.

### 3.3. Permeability models

Different models presented in Table 2 have been used for estimating the relative permeability of nanocomposites. Based on the effect that the clay properties (aspect ratio, orientation and volume fraction), its dispersion and the barrier property of neat polymers have on the gas barrier efficiency of the nanocomposites coatings,^52^ these models considered the aspect ratio, *L*/*W* (with *L*, the length and *W*, the width of a nanoclay platelet), along with the volume fraction (*ϕ*) and the orientation factor (*S*) as parameters affecting tortuosity. In addition to the models presented for polymer nanocomposites, some empirical and analytical models have been proposed in literature to predict the permeability of thin film composite (TFC) membranes and mixed matrix membranes (MMMs) which are mainly extensions of Maxwell model.^53–57^ As an example, a trilayer assembly of CS-PVA-MMT on a PET substrate was studied and the relative permeability, *P*/*P*_0_, was the ratio of the trilayer CS-PVA-MMT permeability coefficient to the one of CS-PVA bilayer on a PET substrate. Only the permeability of the LbL coatings was considered by calculating according to the relationship between the permeability and thickness of a multilayer film:^58^5
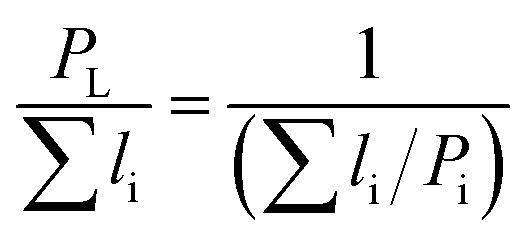
where *P*_L_ stands for the total permeability of a multilayer, *l*_i_ and *P*_i_ are the thickness and permeability for a given layer i respectively.

**Table 2:** Permeability models and their predicted values for filled polymer systems

Model	Relative permeability formula (*P*/*P*_0_)	Considered parameters	Predicted relative permeability
Bharadwaj^60^	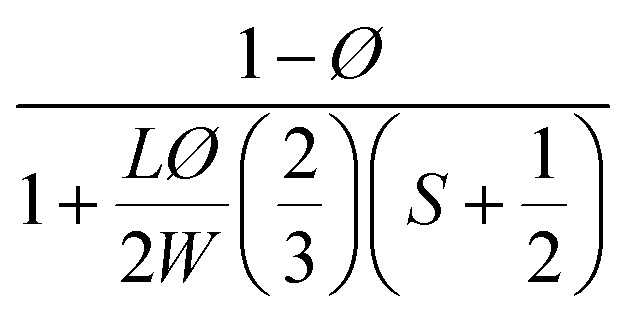	*S* = 1	0.044
		Perfect orientation	
Gusev^61^	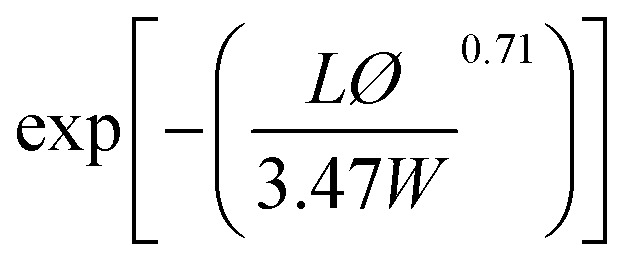	Perfect orientation	0.00296
		No overlapping	
Nielsen^62^	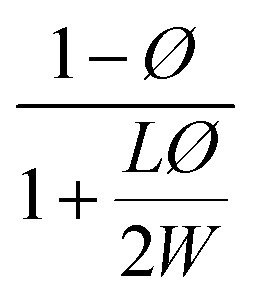	Perfect orientation	0.03448
Cussler^63,64^	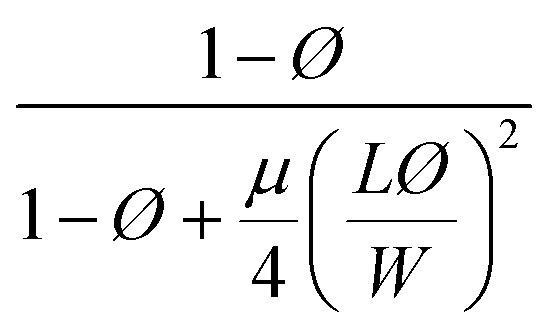	*μ* = 0.5 randomly spaced flakes	0.0009
		*μ* = 0 regularly spaced flakes and infinitely long in one dimension	
Maxwell^65^	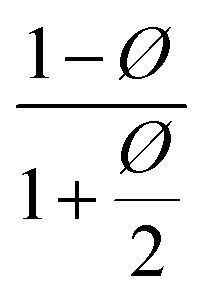	Dilute suspension	0.281

The different nanoclay parameters were obtained experimentally. The representative weight fraction of MMT was determined using TGA as the final residue at 800 °C. The nanoclay volume fraction was calculated according to TGA and profilometry results; and the measured *ϕ* and *f*_CN_ were 0.25 and 0.65 respectively, while a 166 aspect ratio was used in this study.^59^

Among these models, only Bharadwaj didn't consider a perfect orientation. Bharadwaj revised Nielsen's model by introducing the contribution of nanoclay orientation in tortuosity. The order parameter, *S*, in this model is defined as:6
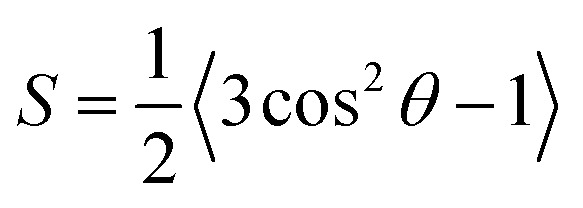
With *θ* as the angle between the direction of the normal unit vectors of the coating and the nanoclay platelets. The angular brackets designate the average for all the clay platelets in the coating.^60^ In his model, Bharadwaj considered three possible orientations, perpendicular where *S* = −1/2, a perfect orientation with *S* = 1 and an intermediate situation representing a random orientation with *S* = 0.

With this model, the lowest achievable *P*/*P*_0_ for this coating is equal to 0.057, when a perfect orientation (*S* = 1) is considered, which is not the case of this LbL coating.

**Fig. 9:**
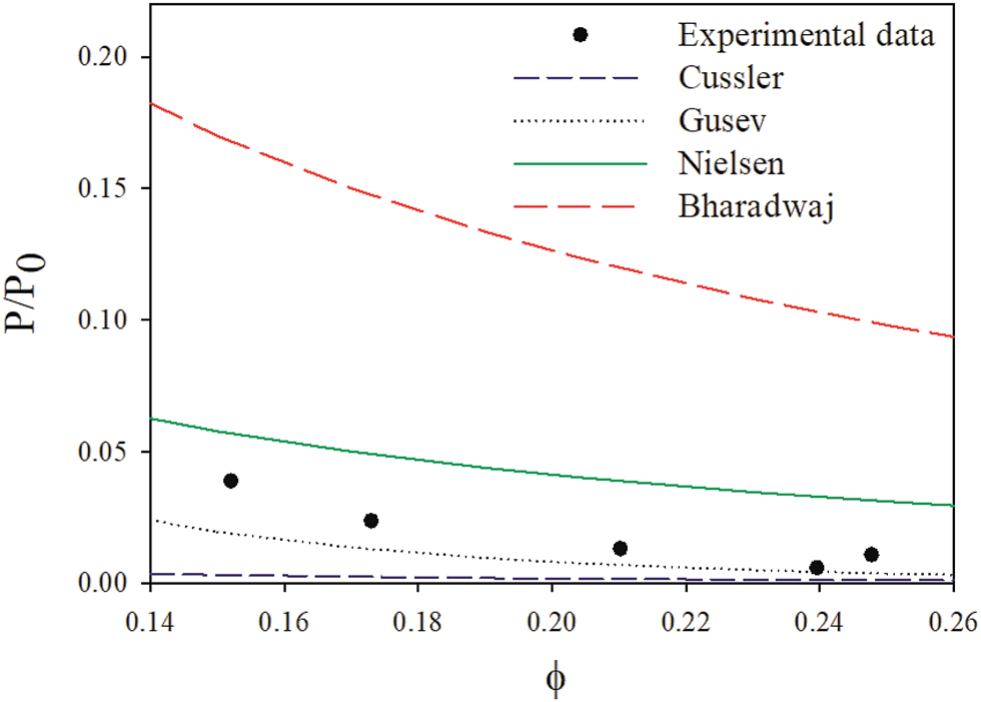
Experimental data compared with permeability models.

With a 25 vol% nanoclay, the measured permeability ratio, *P*/*P*_0_ was 0.00142 (a reduction of 99.85%). Given such a percentage, models considering dilute suspensions such as Maxwell's are the ones to diverge the most from the permeability of LbL deposited coatings. Lower values are obtained with Gusev and Cussler models, since aspect ratio and volume fraction effects on permeability are more accentuated by considering an exponential and a quadratic effect respectively. Gusev's model is, however, mathematical without a physical approach, making thus Nielsen and Cussler models the closest models to the experimental data to be considered, Fig. 9.

**Fig. 10:**
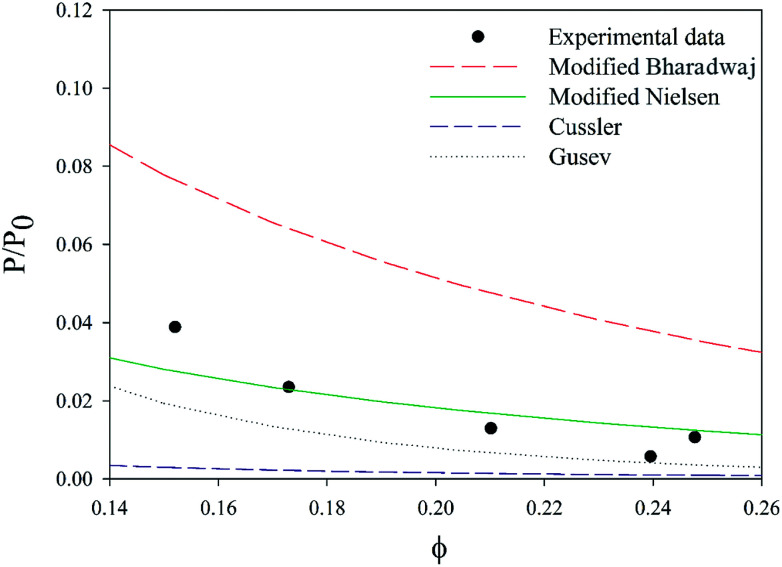
Comparison of the experimental data to various models.

By considering the *P*/*P*_0_ ratio in permeability models, the properties of the matrix are assumed unaltered by the addition of fillers. However, the polymer clay interaction results in an interface region around the clay platelets characterized by a higher density than the bulk.^66^ Due to the high volume fraction of clay in LbL coatings, the volume fraction of this interface becomes considerable. As the studied nanocomposite coatings lack crystallinity, Fig. S5,† impermeable domains are limited to the volume fraction of clay and the interface. The increase of this fraction can be expressed as an increase in the clay volume fraction by a factor *β*. By considering the interfacial region, Nielsen's model can be rewritten as follow:
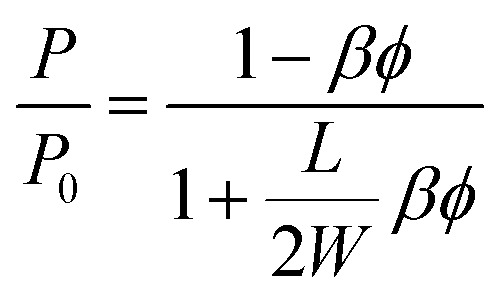


A value of 2, obtained by fitting, for the *β* factor, reduces the RSS from 0.0043 to 0.0015. This same modification for Bharadwaj's model, decreases the RSS ten times (from 0.06 to 0.0058), Fig. 10. This modification can't be applied to Cussler's model as the predicted values are lower than the experimental ones.

## 4 Conclusion

In this work, the orientation of nanoclay platelets in hydrogen bonding based LbL assemblies was investigated and its influence on coatings' properties was discussed in detail. The obtained results highlight that LbL assembled coatings don't show a perfectly oriented nanoclay and its orientation is highly affected by the polymer's physical properties. To have a better understanding of the tortuosity in an LbL film, experimental data were compared to permeability models and the impact of the polymer filler interaction in permeability was highlighted. As the purpose from this study is to have a better understanding of the tortuosity in an LbL film, it was only based on one type of filler. Further work will be conducted to study other types of LbL coatings.

## Conflicts of interest

There are no conflicts to declare.

## Supplementary Material

RA-009-C8RA09522A-s001
