# Correction: Effect of nanoclay orientation on oxygen barrier properties of LbL nanocomposite coated films

**DOI:** 10.1039/c9ra90057h

**Published:** 2019-07-23

**Authors:** Fatma Ben Dhieb, Ebrahim Jalali Dil, Seyed H. Tabatabaei, Frej Mighri, Abdellah Ajji

**Affiliations:** 3SPack NSERC-Industry Chair, CREPEC, Chemical Engineering Department, Polytechnique Montreal C.P. 6079, Succ. Centre Ville Montreal QC Canada H3C 3A7 abdellah.ajji@polymtl.ca; ProAmpac Terrebonne QC Canada J6Y 1V2; CREPEC, Chemical Engineering Department, Laval University Quebec QC Canada

## Abstract

Correction for ‘Effect of nanoclay orientation on oxygen barrier properties of LbL nanocomposite coated films’ by Fatma Ben Dhieb *et al.*, *RSC Adv.*, 2019, **9**, 1632–1641.

The authors regret that the first name of the third author was misspelled in the original article. The correct author names are presented here.

The Royal Society of Chemistry apologises for these errors and any consequent inconvenience to authors and readers.

## Supplementary Material

