# Supplementary material for: Effect of nanoclay orientation on oxygen barrier properties of LbL nanocomposite coated films
Source: RSC Adv. 2019 Jan 11;9(3):1632–41. doi: 10.1039/c8ra09522a (PMC9059654; doi:10.1039/c8ra09522a)
Supplement: RA-009-C8RA09522A-s001 [file RA-009-C8RA09522A-s001.pdf]

Electronic Supplementary Material (ESI) for RSC Advances.
This journal is © The Royal Society of Chemistry 2019

Supporting information

Effect of Nanoclay Orientation on Oxygen Barrier Properties of LbL Nanocomposite Coated Films

Fatma Ben Dhieb[1], Ebrahim Jalali Dil[1], Seyyed H. Tabatabaei[2], Frej Mighri[3] and Abdellah Ajji[1*]

[1] 3SPack NSERC-Industry Chair, CREPEC, Chemical Engineering Department, Polytechnique Montreal, C.P. 6079, Succ. Centre ville, Montreal, QC, Canada H3C 3A7

[2] ProAmpac, Terrebonne, QC, Canada J6Y 1V2

[3] CREPEC, Chemical Engineering Department, Laval University, Quebec, QC, Canada

(*) All correspondence should be addressed to: abdellah.ajji@polymtl.ca

a)

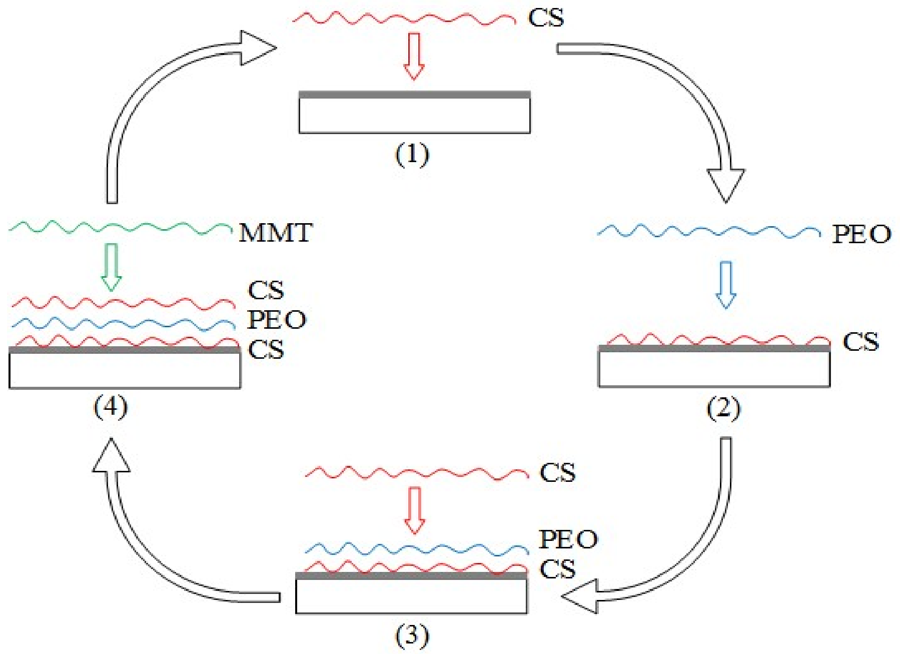

b)

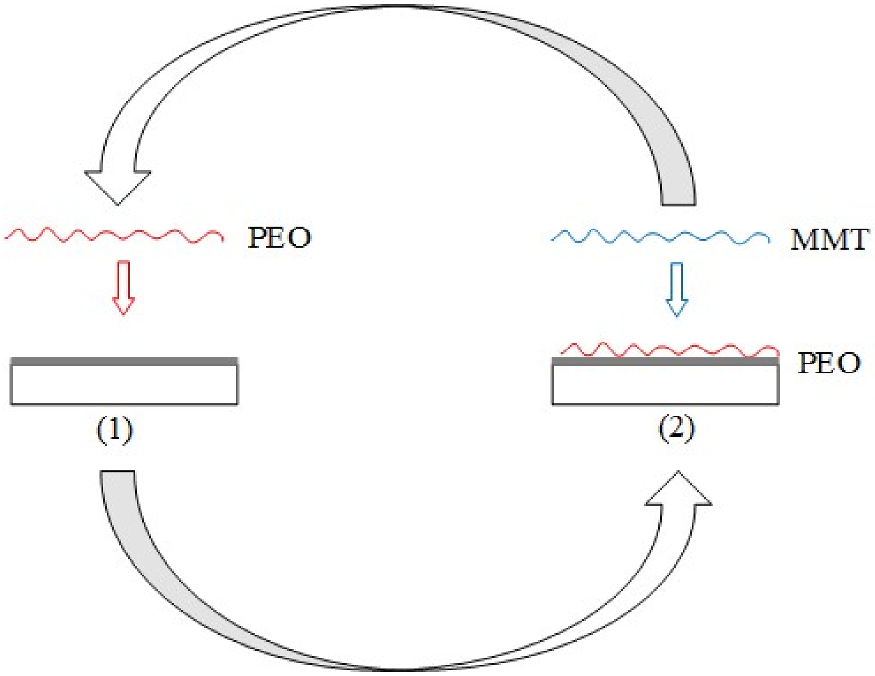

FigureS1: Steps of the deposition of a) one quadlayer chitosan/PEO/chitosan/MMT and b) one bilayer PEO/MMT

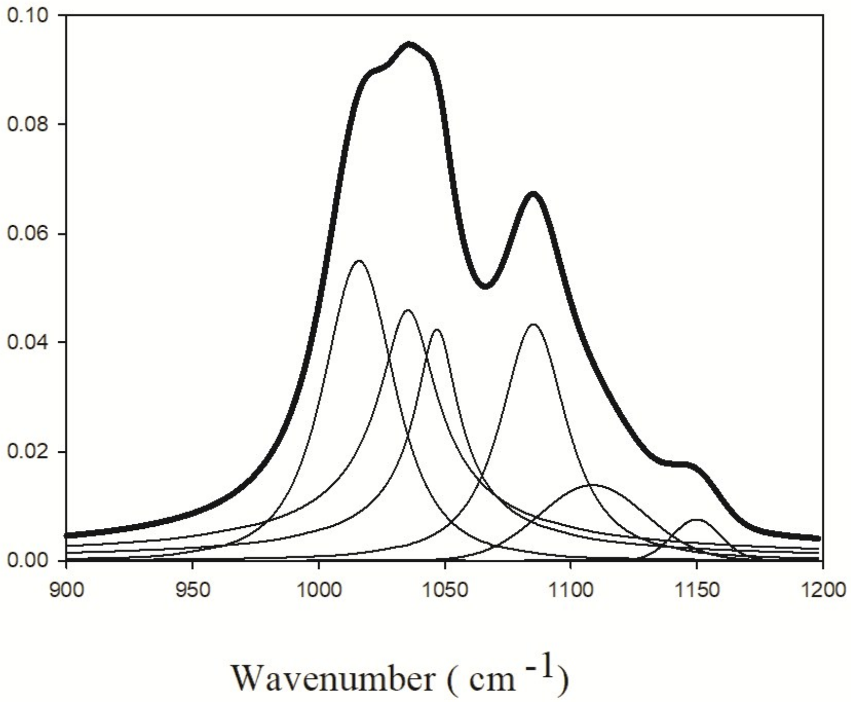

Figure S2: Typical peak deconvolution of the S_0 spectrum for a PVA quadlayers

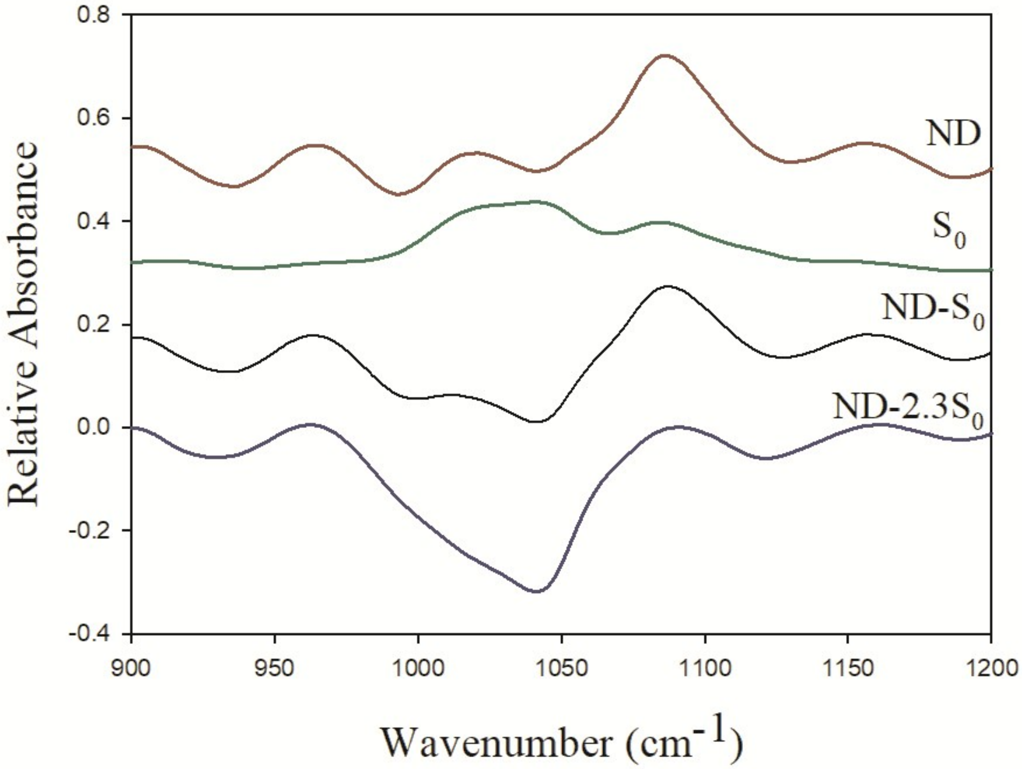

Figure S3: Peak subtraction for a PVP quadlayers

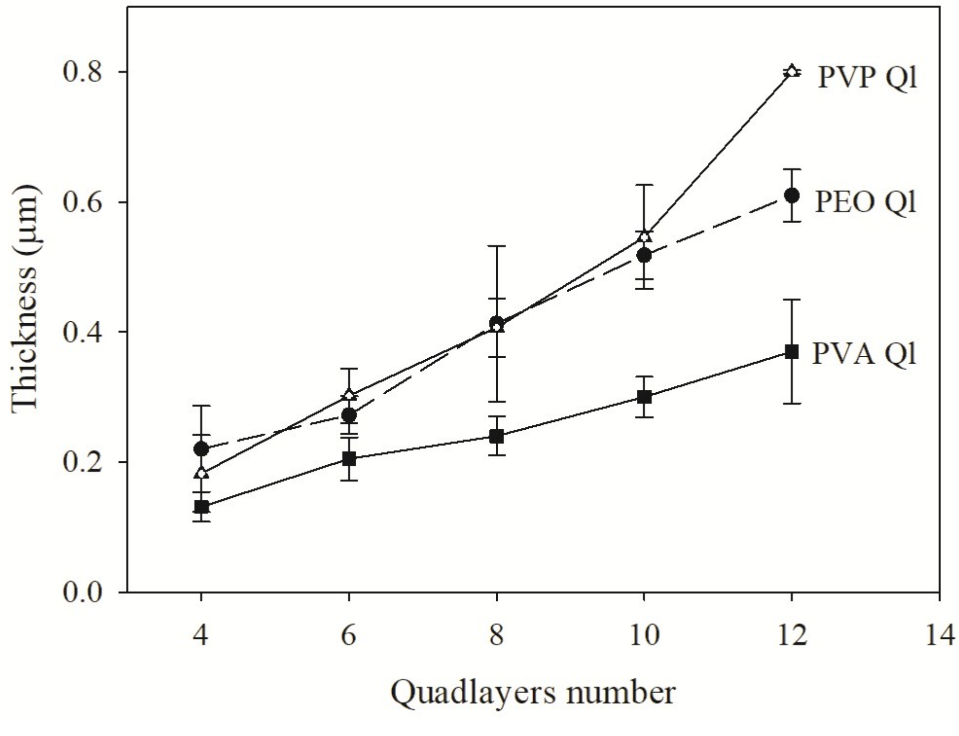

FigureS4: thickness variation with the number of layers for the three assemblies

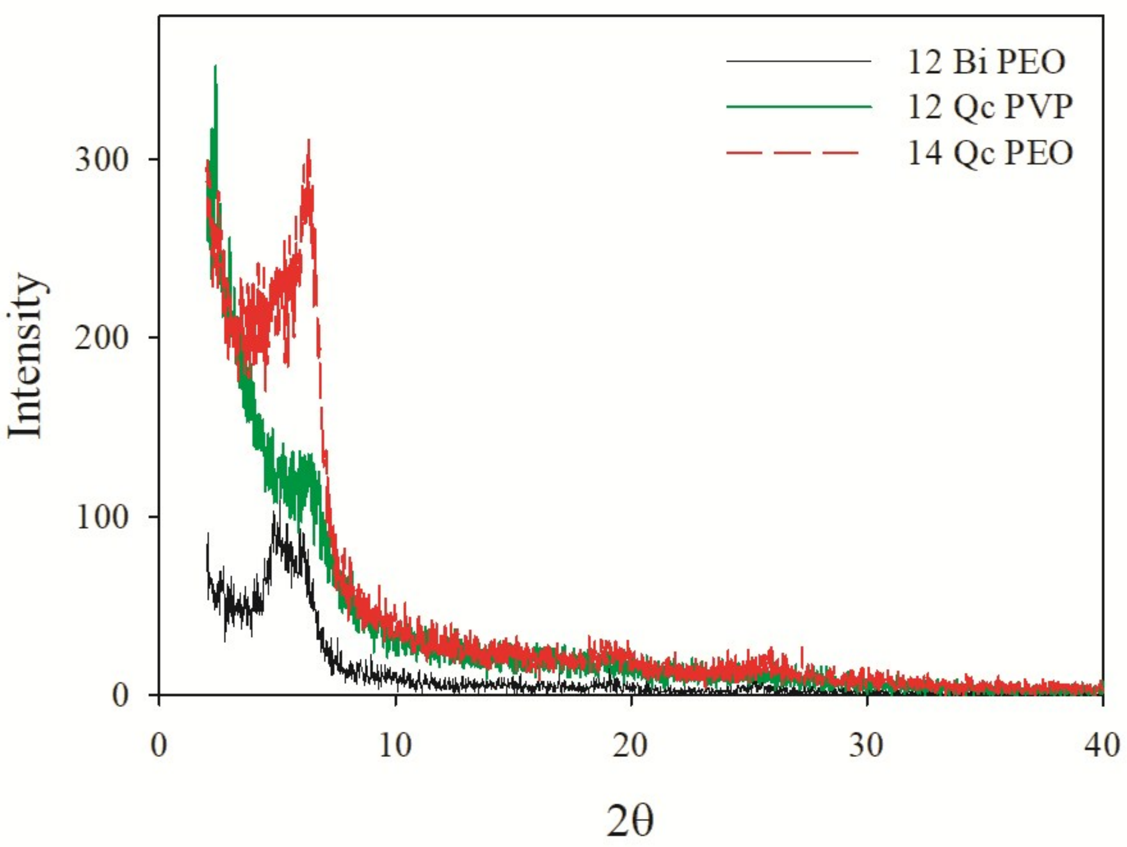

Figure S5: XRD patterns of three of the studied assemblies
